# Dominance of the GI-19 genotype and genomic characterization of the S1 gene in avian infectious bronchitis virus from 2020 to 2024

**DOI:** 10.3389/fcimb.2025.1640152

**Published:** 2025-07-23

**Authors:** Xiaozhen Guo, Cunxia Liu, Feng Hu, Liping Liu, Tong Zhu, Yuehua Gao, Zhongyin Lin, Huaiying Xu, Bing Huang, Zhuoming Qin, Xiuli Ma

**Affiliations:** Institute of Poultry Science, Shandong Academy of Agricultural Sciences, Jinan, Shandong, China

**Keywords:** avian infectious bronchitis virus, S1 gene, recombination, evolutionary rate, cross-protection

## Abstract

The continuous emergence of avian infectious bronchitis virus (IBV) variants poses a critical threat to poultry health and productivity in China. In this study, we conducted comprehensive genetic and antigenic analyses of IBV strains isolated in our laboratory between 2020 and 2024. A total of 94 IBV isolates were sequenced for the S1 gene, revealing widespread nucleotide insertions, deletions, and mutations. Phylogenetic analysis indicated that GI-19 was the predominant genotype (70.21%), followed by GI-13 (21.28%). Recombination analysis using RDP 5.42 identified 14 recombinant strains, primarily GI-13/GI-22 (50%), GI-19/GI-7 (28.6%), and GI-19/GI-22 (21.4%), which were further confirmed using Simplot. Glycosylation analysis revealed that all isolates possessed 14 to18 N-glycosylation sites, whereas only the SDJN3/23 strain contained an O-glycosylation site (position 416). Novel cleavage site motifs (HRRKR, HRHRR, RRFRR) were identified in GI-19 strains, diverging from the canonical HRRRR. The evolutionary rate calculated via BEAST software, was 1.98 × 10^-4^ substitutions/site/year. Serum neutralization assays demonstrated that GI-19 recombinants exhibited partial one-way cross-protection against GI-1, GI-13, and GI-22 genotypes (titer ≥ 1:32), but reciprocal neutralization was limited. Overall, we systematically characterized the genetic diversity and antigenic evolution of the currently circulating IBV strains in China, emphasizing the critical demand for genotype-specific vaccine development and dynamic surveillance systems to counteract viral immune escape.

## Introduction

With the rapid expansion of the poultry industry in China, infectious bronchitis (IB) has emerged as a critical threat to poultry health and productivity (Zeng et al., 2025). As a highly contagious disease, IB primarily targets the respiratory tract, kidneys, and reproductive system, leading to substantial economic losses due to reduced egg production, poor growth performance, and high mortality rates (Cavanagh, 2003). Infectious bronchitis virus (IBV), the causative agent of IB, was first isolated in the United States by Schalk et al. in 1930 and was subsequently identified as the Beaudette strain (Schalk and Hawn, 1931). Currently, IBV distributed worldwide.

IBV, a member of the genus Gamma-coronavirus, *Coronaviridae* family, is a non-segmented, single-stranded positive-sense RNA virus with a genome of approximately 27.6 kb (Jiang et al., 2018). The genome encodes 4 structural proteins (S, N, M, and E), 15 non-structural proteins (nsp2–nsp16), and 4 accessory proteins (3a, 3b, 5a, and 5b) (Rohaim et al., 2020; Lian et al., 2021). The S protein, which is cleaved into the S1 (receptor-binding) and S2 (membrane fusion) subunits, drives viral pathogenicity and host adaptation. The S1 subunit, located in the hypervariable region of the IBV genome, is closely associated with antigenicity, pathogenicity, and tissue tropism. Additionally, it plays a crucial role in inducing neutralizing antibodies and facilitating viral attachment to host cells (Promkuntod et al., 2014; Wickramasinghe et al., 2014). Due to the discontinuous replication of IBV and the lack of proofreading by its RNA polymerase, mutations in the S1 gene generate new serotypes which exhibit no cross-protection with existing ones (Yan et al., 2023). Studies have found that amino acid substitutions in S1 may alter antigenic epitopes, further complicating immune recognition and posing a significant challenge for IBV prevention and control (AR et al., 2019; Moharam et al., 2020).

Although live-attenuated vaccines (e.g., H120, H52, Ma5, 28/86, M41, 4/91, LDT3-A, and QXL87) have been widely used in China, their extensive application has precipitated varying degrees of immune evasion, genetic mutations and recombination events, resulting in the continuous emergence of novel IBV variants.

This study systematically analyzed the genetic evolution, gene recombination, mutation rate, and antigenic cross-reactivity of 94 IBV strains isolated in the laboratory to clarify the differences between the current prevalent and vaccine strains and provide theoretical guidance for the effective prevention and control of IB in the future.

## Materials and methods

### IBV strains

A total of 94 IBV strains isolated from 10 provinces in China, including Shandong, Jiangsu, Liaoning, Heilongjiang, Jilin, Anhui, Hebei, Guangxi, Yunnan, and Shanxi, from 2020 to 2024 were preserved in our laboratory.

### S1 gene sequencing

The S1 gene was amplified using IBV-degenerate primers (forward: 5′-ATGTTGGKRAMRYCWCTD-3′; reverse: 5′-AGTAGAACGTCTAVRACGAY-3′), with a product of approximately 1700 bp. The RT-PCR conditions were as follows: reverse transcription at 45°C for 30 min; initial denaturation at 94°C for 3 min, followed by 35 cycles of denaturation at 94°C for 30 s, annealing at 52°C for 30 s, and extension at 72°C for 90 s, with a final extension at 72°C for 10 min. The RT-PCR products were purified using the Biospin Gel Extraction Kit (BSC02M1A, BioFlux, China) and then were cloned into the pMD18-T vector (6011, Takara, China). The positive samples were sent to Shanghai Shenggong Biological Co., Ltd. for sequencing.

### S1 gene phylogenetic analysis

Nucleotide and amino acid sequence analyses and alignment were performed on the S1 gene of the isolated strain and reference strains of different IBV genotypes downloaded from GenBank using DNAStar software. Following the typing method established by Valastro et al., a phylogenetic tree was constructed using the maximum likelihood method in MEGA 7.0, and 1,000 bootstraps were calculated (Valastro et al., 2016). Genetic distances were calculated, and a neighbor-joining (N-J) evolutionary tree was generated. The potential N-glycosylation and O-glycosylation sites of the S1 gene were analyzed using the online bioinformatics tools NetNGlyc 1.0 Server and NetOGlyc 4.0 Server, respectively (Fan et al., 2019).

### S1 gene recombination analysis

Potential recombination events were evaluated using a dataset of S1 gene sequences, including both isolates and reference strains. Analyses were performed with RDP 5.42 software using seven detection methods (RDP, CHIMAERA, BOOTSCAN, 3SEQ, GENECONV, MAXCHI, and SISCAN). The recombination events were only considered to be significant the following criteria were met: detection by at least five of the seven methods with *P*-values < 1×10^−12^ (Mo et al., 2013). Subsequently, potential recombination events were further verified with Simplot 3.5.1 software (Feng et al., 2018; Jiang et al., 2018).

### Evolutionary rate analysis of the S1 gene

The JModelTest software was used to identify the best model of nucleotide substitution based on 88 candidate models (Darriba et al., 2012), and the optimal model result was GTR+γ+I. A Bayesian tree was constructed using BEAST v1.10.4 software. A Strict molecular clock and Bayesian skyline aggregation model were selected, and the S1 gene was analyzed using the Markov Chain Monte Carlo (MCMC) statistical algorithm. The final effective sample size (ESS) of the operation chain was >200, and the number of operations was 400 million (Zhao et al., 2016). After setting the parameters, BEAST v1.10.4 software was used to generate the xml file and Tracer v1.7.1 was employed to carry out various numerical analyses. The molecular evolution rate of S1 was also confirmed (Baele et al., 2013).

### Cross-serum neutralization test

Representative strains of GI-19, GI-13, GI-22, and GI-1 genotypes were inactivated for vaccine preparation and five 4-week-old SPF chickens were immunized in each group. Booster immunization was conducted 14 days after primary vaccination. Blood samples were collected 14 days after the booster vaccination, and sera were separated and stored at -20°C. The IBV strains of the target genotypes were pre-titrated, and the serum was serially diluted with 100 EID_50_ virus. The mixtures were inoculated into 9-11-day-old SPF embryo (n=5 per dilution). The SPF embryos were observed for 7 days to calculate neutralization titers (highest serum dilution inhibiting ≥50% egg death/dwarfing). Neutralization titers are expressed as log2 values.

### Ethics statement

The SPF chickens were purchased from Shandong Haotai Experimental Animal Breeding Co., Ltd. The experimental protocol was approved by the Ethical Committee for Animals in Shandong Academy of Agricultural Sciences, China.

## Results

### Phylogenetic analysis of IBV

**Figure 1:**
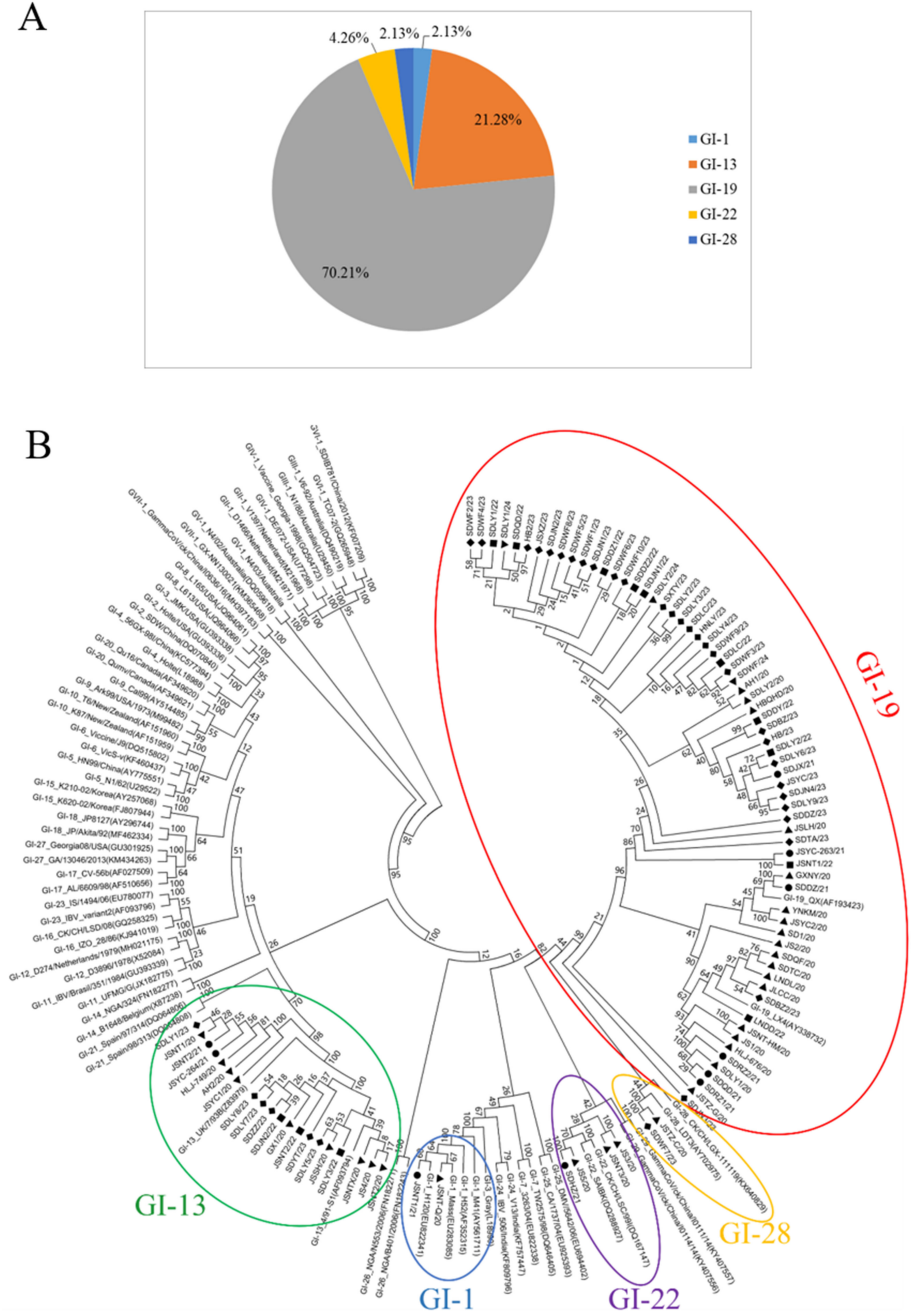
Phylogenetic analysis of isolated IBV strains. **(A)** Genotypes of IBV isolated; **(B)** The phylogenetic tree based on IBV S1 gene. ▲ 2020 ● 2021 ■ 2022 ◆ 2023 ▼ 2024.

To investigate the molecular characteristics of IBV circulating in China, 94 IBV strains were isolated from 10 provinces between 2020 and 2024. The nucleotide and deduced amino acid sequences were systematically aligned against representative reference strains spanning the major genotypes (per the Valastro classification). The homology among the different strains was presented in **Table 1**. The phylogenetic tree was constructed using MEGA 5.0. The 94 IBV isolates were clustered into five GI genotypes: GI-19 (70.21%), GI-13(21.28%), GI-22 (4.26%), GI-28 (2.13%), and GI-1 (2.13%) (**Figure 1A**). Notably, GI-19 was the predominant lineage in all years (**Figure 1B**).

**Table 1:** Genotype-specific homology.

Genotype	Reference Strain	Nucleotide (%)	Amino Acid (%)	Strains (n)
GI-19	LX4	96.0–98.2	89.8–98.0	66
GI-13	4/91	90.1–99.9	88.4–99.8	20
GI-22	CK/CH/LSC/99I	85.0–89.2	82.4–85.3	4
GI-28	LDT3	99.1	98.0	2
GI-1 (Mass)	Mass-type	99.8	99.6	2

### HVRs analysis of the IBV S1 gene

**Figure 2:**
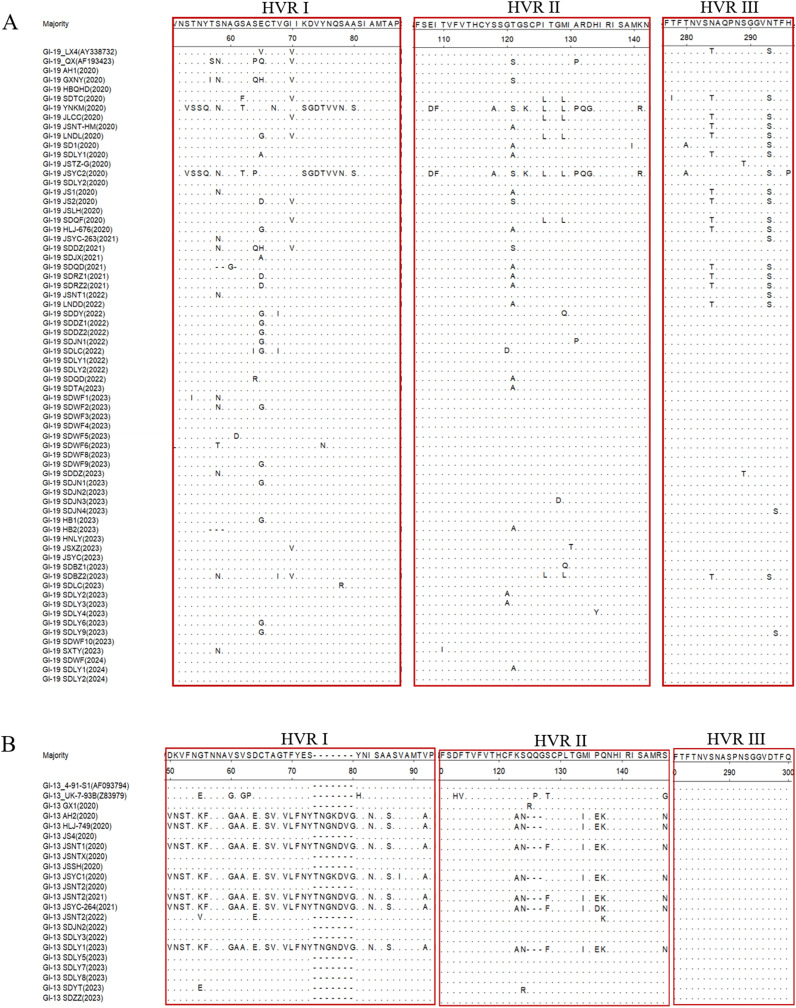
HVRs analysis of the IBV S1 Gene. **(A)** GI-19 isolates; **(B)** GI-13 isolates.

Hypervariable regions (HVRs) may account for the antigenicity and serotype variation and associated with receptor binding. A comparative analysis of the amino acid sequences of the HVRs between the 94 IBV strains and their genotype-matched reference strains was performed using DNASTAR software. The results demonstrated different degrees of amino acid mutations within the HVR, along with sporadic mutations and deletions observed at other sites. Amino acid sequence alignment of the 66 GI-19 isolates with the reference strains LX4 and QX revealed that the amino acid mutations were predominantly located at positions 58, 65, 70, 121, 126, 129, 284, and 293 within S1 (**Figure 2A**). Amino acid sequence alignment between the 20 GI-13 isolates and 4/91 reference strain demonstrated that seven isolates exhibited similar variations, including deletions and point mutations. Notably, these mutations predominantly clustered in HVR I and HVR II (**Figure 2B**).

### Glycosylation site analysis

Analysis of the glycosylation sites of the 94 isolates indicated that only SDJN3/23 exhibited one potential O-glycosylation site at position 416 in the S1 gene and no O-glycosylation sites were predicted in the remaining isolates. All isolates harbored varying numbers of N-glycosylation sites, with significant differences in both quantity and positional distribution across the five genotypes. Compared to the reference strain LX4, all GI-19 isolates (66 isolates) contained 14–18 predicted N-glycosylation sites. Although 52 isolates showed nearly-identical patterns to LX4, 14 isolates displayed only minor positional variations (**Table 2**). The GI-13 genotype (20 isolates) consistently carried 16–17 N-glycosylation sites. Notably, seven isolates shared a novel site (NGSL) at position 143 with reference strain 4/91 (**Table 3**). The remaining 12 isolates largely matched the reference patterns. All 4 GI-22 genotype isolates possessed 16 N-glycosylation sites, compared to the CH/LSC/99I reference strain, uniformly lacking 3 sites: NKTN (position 53), NNST (167), and NVTG (518). Two GI-1 isolates retained 16 N-glycosylation sites, indicating complete conservation with the Mass reference strain. Both JSTZ-C/20 and SD/WF/202307 contained 16 N-glycosylation sites. A shared novel site (NVTN) was identified at position 279 compared with the LDT3 reference strain.

**Table 2:** Number and location of N-glycosylation sites in GI-19 isolates.

Strains	23	52	55	77	104	166	240	279	309	391	428	450	516
LX4	–	NSTN	NYTS	–	NFSE	NFTS	NFSD	–	NLSF	–	NITL	NVTD	–
JSTZ-G/20	+	+	+	–	+	+	+	NVSN	+	–	+	+	–
YNKM/20	NDTY	+	+	NASS	+	+	+	+	+	–	+	+	–
JSYC2/20	NDTY	+	+	NASS	+	+	+	+	+	–	+	+	–
SDDZ/21	–	+	+	–	+	+	+	NVSN	+	–	+	+	–
JSNT1/22	–	+	+	–	+	+	+	NVSN	+	–	+	+	NETG
SDLY3/23	–	+	+	–	+	+	+	NVSN	–	–	+	+	–
SDJN1/23	–	+	+	–	+	+	–	NVSN	–	NYSK	+	+	–
SDJN2/23	–	+	+	–	+	+	+	NVSN	–	–	+	+	–
SDJN3/23	–	+	+	–	+	–	+	NVSN	–	–	+	+	–

+ Represent the same as the reference strain;

- Represent deletion.

**Table 3:** Number and location of N-glycosylation sites in GI-13 isolates.

Strains	54	75	103	143	180	214	239	427	449	456	515	532
GI-13 4/91	NGTN	NISA	NFSD	–	NETT	NGTA	NFSD	NITL	NVTE	–	NETD	NGTR
JSNT1/20	+	+	+	NGSL	+	+	+	+	+	–	–	+
HLJ-749/20	+	+	+	NGSL	+	+	+	+	+	–	+	+
AH2/20	+	+	+	NGSL	+	+	+	+	+	–	+	+
JSYC1/20	+	+	+	NGSL	+	+	+	+	+	–	+	+
JSYC-264/21	+	+	+	NGSL	+	+	+	+	+	–	–	+
JSNT2/21	+	+	+	NGSL	+	+	+	+	+	–	+	+
SDLY1/23	+	+	+	NGSL	+	+	+	+	+	–	–	+
SDLY8/23	+	+	+	–	+	+	–	+	+	–	+	+

+ represent the same as the reference strain;

- represent deletion.

### Cleavage site analysis

The cleavage sites of all isolates were identified, and the results showed that there were 5 cleavage site sequences: HRRRR (58 isolates, 61.7%), RRSRR (20 isolates, 21.28%), HRHRR (7 isolates, 7.45%), RRFRR (5 isolates, 5.3%), and HRRKR (4 isolates, 4.26%). The cleavage site of the S protein of GI-19 (56/66) isolates was the same as that of the reference LX4 (HRRRR), however, three variants were observed: HRRKR, HRHRR, and RRFRR. The cleavage sites of the 20 GI-13 isolates were the same as those of the reference strain 4/91 (RRSRR). The cleavage sites in the four GI-22 isolates were HRRRR and HRRKR. Both GI-1 and GI-28 exhibited uniform RRFRR. Details of the S1 sequence lengths and cleavage site distributions of the isolates are shown in **Table 4**.

**Table 4:** Length of the S1 sequence and the distribution of cleavage sites.

Genotype	S1 length	Number	Genotype	Cleavage sites	Number
GI-19	1620bp/540aa	61	GI-19	HRRRR	56
	1605bp/535aa	1			
	1608bp/536aa	1		HRRKR	2
	1611bp/537aa	1			
	1614bp/538aa	1		HRHRR	7
	1623bp/541aa	1			
GI-13	1617bp/539aa	12		RRFRR	1
	1614bp/538aa	1	GI-13	RRSRR	20
	1626bp/542aa	7			
GI-22	1608bp/536aa	1	GI-22	HRRRR	2
	1611bp/537aa	1		HRRKR	2
	1632bp/544aa	2	GI-28	RRFRR	2
GI-28	1620bp/540aa	2	GI-1	RRFRR	2
GI-1	1611bp/537aa	2			

### Recombination analysis of the IBV S1 gene

**Figure 3:**
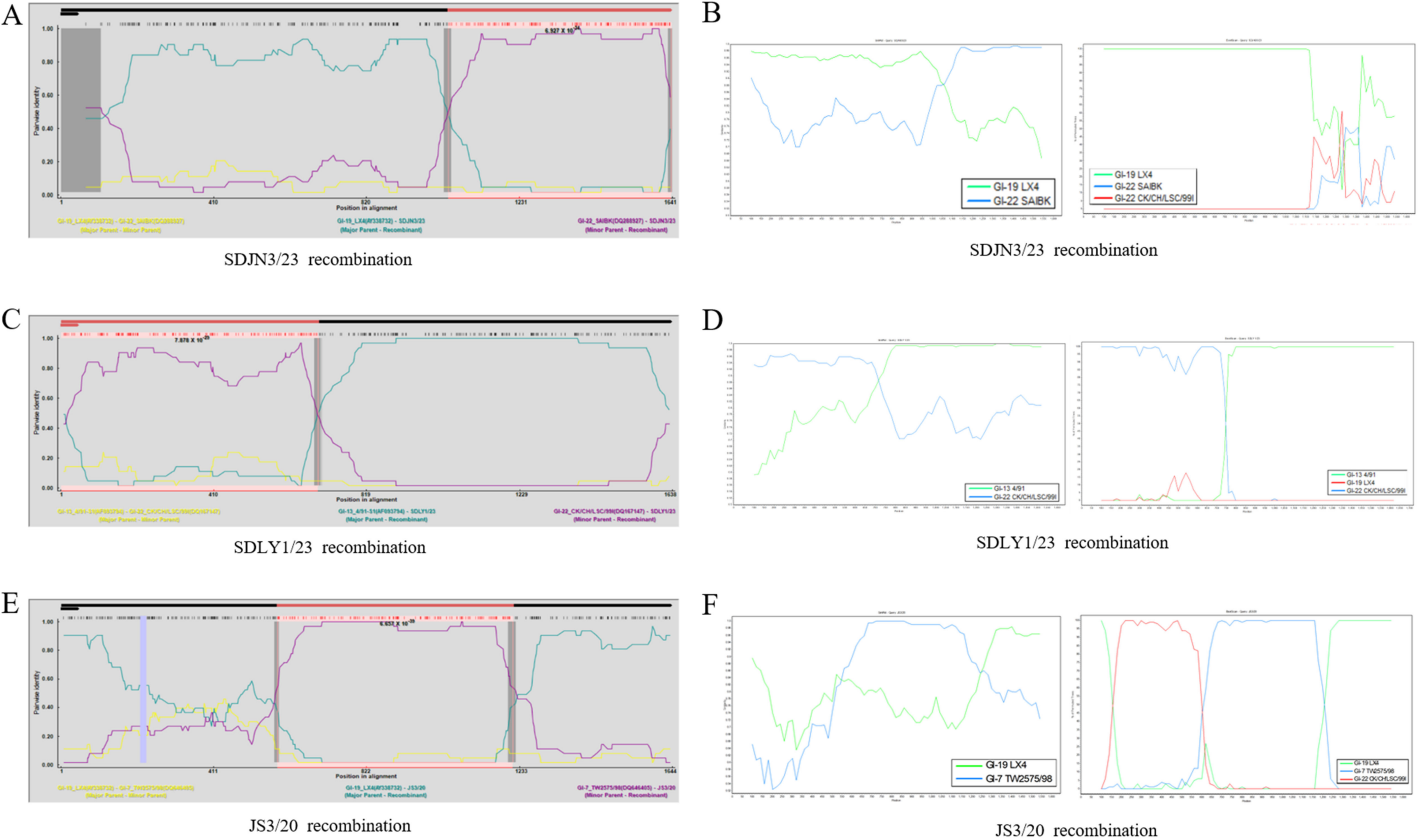
Recombination analysis of the IBV S1 gene. **(A, C, E)** SDJN3/23, SDLY1/23, JS3/20 recombination identified by RDP 5.42, respectively. **(B, D, F)** SDJN3/23, SDLY1/23, JS3/20 recombination identified by Simplot, respectively.

Recombination analysis revealed that 14 of the 94 IBV isolates (14.89%) exhibited statistically significant recombination events in S1 (**Table 5**), as detected by RDP 5.42 (*P* < 0.05). The predominant recombinations involved the GI-13/GI-22 (7/14), GI-19/GI-7 (4/14), and GI-19/GI-22 (3/14) genotypes. To validate the recombination events identified by RDP 5.42, three representative strains (SDJN3/23, SDLY1/23, and JS3/20) exhibiting distinct genotype combinations were selected for in-depth analysis using Simplot v3.5.1 (**Table 6**). These results indicated that SDJN3/23 was a recombinant strain derived from the parental strains GI-19 LX4 and GI-22 SAIBK, with a breakpoint at position 1102bp. SDLY1/23 was derived from parental strains GI-13 4/91 and GI-22 CK/CH/LSC/99I with a breakpoint at position 720 bp. JS3/20 was derived from the parental strains GI-19 LX4 and GI-7 TW2575/98, with breakpoints at position 604 bp and 1288 bp (**Figure 3**). These findings are consistent with those of the RDP 5.42 analysis, confirming the occurrence of recombination events in these isolates.

**Table 5:** Genetic recombination events of the S1 gene of IBV isolates detected by RDP 5.42 software.

Potential recombinant	Breakpoints		Major parent^a^			Minor parent^b^			*P* Value^c^
	Beginning	Ending	Strain	Genotype	Similarity	Strain	Genotype	Similarity	
JSNT1/20	1	720	UK/7/93B	GI-13	99.3%	CH/LSC/99I	GI-22	95.6%	8.854×10^-37^
HLJ-749/20	1	720	UK/7/93B	GI-13	99%	CH/LSC/99I	GI-22	95.8%	1.602×10^-25^
AH2/20	1	720	UK/7/93B	GI-13	99%	CH/LSC/99I	GI-22	95.9%	1.804×10^-26^
JS3/20	604	1288	QX	GI-19	89.1%	TW2575/98	GI-7	99.1%	1.035×10^-35^
YNKM/20	1	668	LX4	GI-19	96.3%	TW2575/98	GI-7	98.2%	2.867×10^-30^
JSYC1/20	1	720	UK/7/93B	GI-13	99%	CH/LSC/99I	GI-22	95.9%	9.925×10^-27^
JSNT3/20	604	1288	QX	GI-19	89.1%	TW2575/98	GI-7	98.9%	5.429×10^-30^
JSYC2/20	1	752	QX	GI-19	97%	TW2575/98	GI-7	98.1%	1.898×10^-31^
JS5/20	120	1393	LX4	G1-19	97.3%	CH/LSC/99I	GI-22	91.7%	9.699×10^-5^
JSYC-264/21	720	1772	CH/LSC/99I	GI-22	95.5%	UK/7/93B	GI-13	99.3%	1.545×10^-34^
SDHZ/21	138	1393	LX4	GI-19	94.3%	CH/LSC/99I	GI-22	92.2%	1.410×10^-17^
JSNT2/21	720	1710	CH/LSC/99I	GI-22	95.1%	UK/7/93B	GI-13	97.1%	3.791×10^-28^
SDLY1/23	1	720	4/91	GI-13	99.5%	CH/LSC/99I	G1-22	95.3%	7.878×10^-29^
SDJN3/23	1	1102	LX4	G1-19	96.6%	SAIBK	GI-22	99%	6.927×10^-34^

a Major parent, the parental strain that provides the larger fragment sequence.

b Minor parent, the parental strain that provides the smaller fragment sequence.

c The *P* value was calculated by RDP 5.42.

**Table 6:** P values of different methods in each recombination event.

Potential recombinant	Major parent	Minor parent	The *P* value of seven methods in each recombination event						
			RDP	GENECONV	Bootscan	MaxChi	Chimaera	SiScan	3Seq
SDJN3/23	GI-19 LX4	GI-22 SAIBK	6.927×10^-34^	4.757×10^-28^	8.779×10^-31^	6.312×10^-21^	1.962×10^-20^	4.251×10^-27^	6.296×10^-53^
SDLY1/23	GI-13 4/91	GI-22 CK/CH/LSC/99I	7.878×10^-29^	2.806×10^-20^	8.361×10^-20^	9.544×10^-25^	5.430×10^-22^	6.730×10^-21^	1.696×10^-60^
JS3/20	GI-19 LX4	GI-7 TW2575/98	6.657×10^-39^	3.867×10^-34^	4.040×10^-32^	2.939×10^-24^	1.812×10^-24^	9.614×10^-25^	3.151×10^-41^

### Evolutionary rate analysis of the IBV S1 gene

The evolutionary rate of the remaining 80 isolates was analyzed after excluding 14 recombinant strains. The optimal model as GTR+γ+I was selected using JModelTest software, and estimated under a strict molecular clock with Bayesian skyline coalescent model. The mean nucleotide substitution rate of the isolated S1 gene was 1.98×10–^4^ substitutions/site/year, and the 95% HPD interval was 1.55-2.46×10^-4,^ as estimated using BEAST software (**Table 7**).

**Table 7:** Evolutionary rate of IBV calculated using BEAST software.

Gene	Substitution/evolutionary rate (substitutions/site/year)	
	Rate	95%HPD
S1	1.98×10^-4^	1.55-2.46×10^-4^

### Serum cross neutralization test

Cross-neutralization tests were conducted between the main genotypic representative isolates SDJN3/23 (GI-19), JS3/20 (GI-22), and SDLY1/23 (GI-13), and the classical vaccine strain H120 (GI-1). The results showed that JS3/20 had the highest self-neutralization titer (1:256), but minimal cross-reactivity with GI-19/GI-13/GI-1 sera (<1:8). The SDJN3/23 strain, isolated in 2023, exhibited a strong self-neutralization titer (1:128) and demonstrated good cross-neutralization with GI-13, GI-1 and GI-22 sera (≥1:32) (**Table 8**).

**Table 8:** Viral cross-neutralization tests.

serum strain	SDJN3/23 (GI-19)	JS3/20 (GI-22)	SDLY1/23 (GI-13)	H120 (GI-1)
SDJN3/23 (GI-19)	128	32	64	64
JS3/20 (GI-22)	16	256	<8	<8

## Discussion

Since its isolation in the United States in 1930, IBV has emerged as a major threat to the global poultry industry, significantly impairing the production performance of both broiler and layer chickens and causing substantial economic losses. In recent years, the widespread use of live-attenuated vaccines has increased immunological pressure, facilitating continuous recombination between wild-type and vaccine strains, ultimately leading to the emergence of novel variants and escalating challenges in disease control (Zeng et al., 2025). In this study, 94 IBV strains were successfully isolated from suspected IBV-infected chicken flocks in China between 2020 and 2024. Through genetic evolutionary analysis of the S1 genes, we aimed to provide critical insights for predicting and managing IB outbreaks in China.

The IBV S gene exhibits high genetic plasticity, with amino acid variations predominantly occurring in the S1 subunit. Minor changes in S1 can lead to the emergence of new genotypes or serotypes, resulting in antigenically distinct strains that can evade vaccine-induced immunity (Ma et al., 2019). Lee et al. mapped key mutation hotspots within the HVRs of the S1 gene (Lee et al., 2008; Shan et al., 2018; Ren et al., 2020). In this study, sequence analysis of 94 IBV isolates revealed widespread nucleotide insertions, deletions, and point mutations in the HVRs of S1, with the highest mutation frequency observed in GI-19 lineage strains. Given that these mutations occur in critical antigenic domains, further investigation is warranted to determine their effects on viral pathogenicity, infectivity, and immune evasion mechanisms.

Genetic recombination plays a pivotal role in the evolution of IBV and often leads to the emergence of novel strains through interspecific recombination events. This process significantly amplifies the genetic diversity and complexity of IBV populations, complicating disease control efforts (Ma et al., 2019; Shosha et al., 2025; Wang et al., 2025). Here, 14 recombinant variants were identified among the 94 isolated strains, with the following genotype-specific patterns: GI-13/GI-22, GI-19/GI-7, and GI-19/GI-22. These findings underscore the extensive recombination complexity of IBV circulating in China, with greater uncertainty at recombination sites. Notably, GI-13 and GI-19 exhibited a heightened recombination propensity, with GI-19 showing a particularly unpredictable breakpoint distribution. This may explain the epidemiological predominance of these genotypes, especially GI-19.

The sequence of cleavage recognition sites may reflect geographically distinct viral evolutionary patterns (Jackwood et al., 2001). The functional implications of these variations among the different genotypes, particularly their potential influence on viral virulence, tissue tropism, or host adaptation, warrant further mechanistic investigation. Emerging evidence has suggested that viral glycosylation sites may be correlated with nephropathogenicity and modulate critical functions, including S protein-mediated membrane fusion, viral infectivity, and replication efficiency (Zheng et al., 2018; Parsons et al., 2019; Mase et al., 2022). Comprehensive glycosylation site analysis demonstrated that only the SDJN3/23 strain exhibited a predicted O-glycosylation site at position 416 and that all isolates contained 14–18 predicted N-glycosylation sites. Additionally, N-glycosylation patterns were genotype-specific, with GI-19 showing the highest variability. These findings highlight the genotype-dependent glycosylation patterns in circulating IBV strains. The functional consequences of these variations, particularly regarding S1 protein mediated viral tropism, tissue specificity, and infectivity, require further mechanistic investigation through reverse genetics and *in vivo* studies.

Under the optimal model, the mean nucleotide substitution rate was 1.98 × 10^-4^ substitutions/site/year for the S1 gene of IBV isolates from 2020 to 2024 using the Bayesian method, which was higher than that of the isolates collected from 1985 to 2017 in China (Fan et al., 2019). These findings demonstrate significantly accelerated evolutionary dynamics in contemporary IBV populations, likely driven by increased immune selection pressures. The Bayesian approach is particularly valuable for tracking rapid pathogen evolution under non-clockwise conditions.

The lack of standardized positive reference sera for IBV neutralization tests in China has posed a major challenge to reliable serotype classification (Chen et al., 2017). Previous studies have suggested a potential correlation between genotype and serotype (Wang and Huang, 2000; Xiong et al., 2024). In this study, phylogenetic analysis revealed a close evolutionary relationship between the GI-19 and GI-22 genotype branches, suggesting that their serotypes might be related. To systematically assess the antigenic variation, four genotype-representative strains were selected for monovalent serum production and comprehensive cross-neutralization assays. The results demonstrated partial cross-reactivity between JS3/20 (GI-22) and SDJN3/23 (GI-19), whereas no cross-reactivity was observed between JS3/20 and strains of GI-1 or GI-13 genotypes. This phenomenon may be attributed to the recombinant origin of JS3/20, which is derived from the GI-7 and GI-19 genotypes. Notably, the SDJN3/23 isolate (GI-19) exhibited varying degrees of unidirectional neutralization and protection in all four sera, suggesting that this strain possesses a broader antigenic spectrum than the others. This enhanced cross-reactivity may be linked to the dual recombinant background (GI-19 × GI-22).

## Conclusions

This study systematically characterized 94 IBV isolates by analyzing the genetic variation, cleavage and glycosylation site polymorphisms, recombination and evolutionary rate of the S1 gene. The data indicated widespread nucleotide variations in the S1 genes across recent Chinese isolates, along with the co-circulation of multiple genotypes, underscoring the dynamic genetic evolution of IBV and the necessity for enhanced surveillance. In addition, the potential of co-infection by two or more different IBV strains within the same sample should be pay more attention in subsequent virus detection and isolation. To improve disease control, it is crucial to integrate local epidemiological data for vaccine selection and accelerate the development of multivalent or universal epitope-targeted vaccines.

## Data Availability

The raw data supporting the conclusions of this article will be made available by the authors, without undue reservation.
